# Facing the FACS—Using AI to Evaluate and Control Facial Action Units in Humanoid Robot Face Development

**DOI:** 10.3389/frobt.2022.887645

**Published:** 2022-06-14

**Authors:** Marius Auflem, Sampsa Kohtala, Malte Jung, Martin Steinert

**Affiliations:** ^1^ TrollLABS, Department of Mechanical and Industrial Engineering, Faculty of Engineering, Norwegian University of Science and Technology (NTNU), Trondheim, Norway; ^2^ Robots in Groups Lab, Department of Information Science, Cornell University, Ithaca, NY, United States

**Keywords:** humanoid robots, artificial intelligence, medical simulation, robot development, facial action units, facial expression

## Abstract

This paper presents a new approach for evaluating and controlling expressive humanoid robotic faces using open-source computer vision and machine learning methods. Existing research in Human-Robot Interaction lacks flexible and simple tools that are scalable for evaluating and controlling various robotic faces; thus, our goal is to demonstrate the use of readily available AI-based solutions to support the process. We use a newly developed humanoid robot prototype intended for medical training applications as a case example. The approach automatically captures the robot’s facial action units through a webcam during random motion, which are components traditionally used to describe facial muscle movements in humans. Instead of manipulating the actuators individually or training the robot to express specific emotions, we propose using action units as a means for controlling the robotic face, which enables a multitude of ways to generate dynamic motion, expressions, and behavior. The range of action units achieved by the robot is thus analyzed to discover its expressive capabilities and limitations and to develop a control model by correlating action units to actuation parameters. Because the approach is not dependent on specific facial attributes or actuation capabilities, it can be used for different designs and continuously inform the development process. In healthcare training applications, our goal is to establish a prerequisite of expressive capabilities of humanoid robots bounded by industrial and medical design constraints. Furthermore, to mediate human interpretation and thus enable decision-making based on observed cognitive, emotional, and expressive cues, our approach aims to find the minimum viable expressive capabilities of the robot without having to optimize for realism. The results from our case example demonstrate the flexibility and efficiency of the presented AI-based solutions to support the development of humanoid facial robots.

## 1 Introduction

Humanoid robots with expressive attributes that encourage social interactions with humans are an important topic for various fields of research and industrial contexts (Breazeal, 2009). With recent technological advancements pushing the boundaries for complex behavior and humanlike appearance, several robots designed to look and behave like humans have been developed (Becker-Asano and Ishiguro, 2011; Ameca 2021; Cominelli et al., 2021; Sophia 2021). New materials, accessible electronics, rapid prototyping, and artificial intelligence (AI) have all been key enabling factors for the emergence of these uncannily realistic (humanlike) robots (Oh et al., 2006). Not only are they approaching a realistic visual resemblance to humans (Mori et al., 2012), but by movements and simulated cognition, robots are enabling eerily realistic interactions with people (Pan et al., 2020). Hence, how the robot looks, behaves, and reacts are important aspects to consider when designing solutions for human-robot interaction applications (Cameron et al., 2018; Ghazali et al., 2018). In this context the face of the robot is particularly important for non-articulate responses like body language, expressions, and sudden reactions. The synergetic effects of realistic appearance and complex humanlike behavior, i.e., gaze, expressions, and motor abilities, have been identified as essential factors (Minato et al., 2004). Hence, novel robots with expressive capabilities have facilitated research on mimicking, synthesizing, and modelling of robotic face movements (Wu et al., 2009; Magtanong et al., 2012; Mazzei et al., 2012; Meghdari et al., 2016). Furthermore, researchers aim at providing insights on how we evaluate, recognize, respond, react, and interact with such social and emotional humanlike robots (Hofree et al., 2018; Hortensius et al., 2018; Jung and Hinds, 2018; Tian et al., 2021).

However, while advanced expressive robots enable us to explore ways to achieve humanlike face movements, there is a lack of tools and methods supporting such robots’ (early-stage and ongoing) development. Specifically, accessible, fast, and easy-to-use tools and methods aiding in prototype evaluation and control of new humanoid robots. Furthermore, these tools should not be limited to specific hardware architecture and should moreover, provide objective feedback on obtainable face movement to the designers. For example, characterizing the relations between actuator input and resulting face movement could be critical to understanding (and improving) humanoid robots’ design. Such tools may enable simplified control of these robots by using human face parameters, such as facial action units (AUs), to create a variety of custom facial responses and expressions (Ekman and Friesen, 1978). For evaluating generated expressions or movement, automatic visual inspection leveraging human face tracking software and AI applications could be purposeful to mitigate designer (or user) biases. Additionally, this could speed up learning the potentials and limitations of hardware prototypes, given that different use-cases yield different design constraints and needs for future robots. Hence, resources that inform development of humanoid robots are essential as these will become custom in a variety of industrial contexts.

This paper presents the use of open-source computer vision and machine learning (ML) methods as tools for supporting the development and evaluation of robotic faces. The approach of utilizing these tools is showcased in a development project of a new humanoid robot with facial movement capabilities intended for healthcare learning applications. The development project has utilized a highly iterative approach for designing, building, and testing prototypes. This is an effective way of dealing with ambiguity caused by complex design problems before functional requirements have been established. However, a challenge with prototyping a robotic face is the unavoidable subjective effects of designing for social and communicative attributes such as face appearance, movement, and subsequently, expressions. As both user and designer biases are evident, a flexible method for rapidly evaluating prototypes and informing design decisions is needed. AI is a broad field that includes computer vision and machine learning algorithms that can make decisions on a par with humans. Hence, we present a method for utilizing open and readily available AI-based software solutions without requiring specialized hardware or human-in-the-loop for obtaining facial action units from random motor movements to create a control model for the robot. Furthermore, the generated data are used to inform the current robot design by using AU correlations, both between other AUs and against motor actuation modes. The presence or absence of AUs in the obtained data is used as a performance measurement for the prototype capabilities. The proposed method is also applicable for any type of mechanically actuated robotic face resembling a human, regardless of the number of control units or face characteristics.

This is a proof-of-concept intended to showcase the applicability of using available AI tools for design of humanoid robots, and the advantages and limitations of using such tools during development. Furthermore, we want to give an outlook and highlight the potential benefits of using this method in development of flexible and customizable humanoid robots for healthcare learning applications through a development case example. To summarize, the aim of our method is to 1) gain objective and actionable insights for early-stage robotic face development, 2) rapidly generate AU data and train a control model tailored to the specific robot face appearance and actuation capabilities, and 3) to objectively evaluate the current robot design and control model using various datasets.

## 2 Background

### 2.1 Robot Motion and Behavior

State-of-the-art robots designed to look and behave like humans are complex (and expensive) equipment, encompassing delicate hardware to achieve many degrees of freedom for facial movement (Faraj et al., 2020). Expressive motions and behavior are important as they can communicate the internal state of the robot and convey information about its’ affect, fatigue, intent, style, and personality (Venture and Kulić, 2019). Expressive robots are often developed by inspiration from the anatomy of real humans using biomimicry (Hanson et al., 2002; Pioggia et al., 2004; Hashimoto et al., 2006) or by mechanical modeling of target output movements predetermined from human face capabilities (Magtanong et al., 2012). Subsequently, control and evaluation of such robots becomes challenging utilizing manual operation and hand-coded motion sequences (Venture and Kulić, 2019). Moreover, due to the complex system interactions and dynamic behavior, such robots may need to be analyzed after being fully developed (Ishihara et al., 2021). Therefore, exploring new and intuitive ways to control (and evaluate) humanoid robotic faces has been a topic of interest. For example, to control using machine vision software and AI to recognize specific human expressions and mirroring, recreating, or reacting using a robotic agent (Kim et al., 2006; Silva et al., 2016; Todo, 2018). Similarly, AI applications have been utilized to analyze robots’ facial capabilities and automatically learn various expressions (Wu et al., 2009; Mazzei et al., 2012; Meghdari et al., 2016; Chen et al., 2021; Rawal et al., 2022). For automated control of robotic faces, using AUs is valuable as it becomes a transferal unit of facial movement, representing both the human action and the robot’s actuation capabilities (Lin et al., 2011; Lazzeri et al., 2015; Faraj et al., 2020). However, limitations of these approaches include the sequential development of robot and control systems, thus restricting rapid design cycles and performing simultaneous and cross-disciplinary improvements. Furthermore, the tools and methods deployed are often restricted to a set of static expressions or are limited to specific hardware or appearance, making them less suited in the early, conceptual, and prototype-driven development of humanoid robots. The novelty of our approach compared to existing solutions is the possibility of effortlessly capturing AUs from different robot designs with varying degrees of freedom and using this information to support the development process and to rapidly create control models for expression synthesis. While Wu et al. (2009) used the correlations between AUs and servos of a high degree of freedom robot face to create a linear mapping between the two, we highlight the importance of additionally using AU to AU correlation analysis to gain valuable information and support the development of expressive robots. We also show how modern and scalable ML algorithms can quickly approximate both linear and non-linear AU to servo mappings with limited degrees of freedom.

### 2.2 Case of a Medical Simulator Face With Expressive Capabilities

Simulation-based medical training and education is an area where humanlike robots already play an important role. In this context, the robot, often referred to as the manikin, portrays a patient that needs care and treatment. Having evolved from static and limited anatomical chassis’, manikins have gained a range of simulated physiological and cognitive abilities enabled by remote-controlled operation, and autonomous or semi-autonomous control systems (Cooper and Taqueti, 2008). These manikins have excided far beyond their initial use-cases of psychomotor skills training and routine practice for medical students. However, the non-articulate communicative aspects of such robots remain limited, often having a generic and static appearance incapable of performing facial movements to render expressions, communicate, react, or simulate important medical cues (Lalitharatne et al., 2020). For training scenarios where medical simulators (i.e., robots) are used instead of real patients, the simulators should accommodate multimodal tasks, such as combining data acquisition, interventions, and clinical assessment (Pourebadi and Riek, 2018). This would enable the simulated patient’s facial movements and behavior to be observed and used for evaluating medical conditions, cognitive abilities, and emotional states to pose a diagnosis. Furthermore, the standardized appearance of simulators is limiting, as it is important to capture various patient characteristics to reflect the diversity found in the general population. Age, gender, ethnicity, and cultural traits should therefore be adequately captured by robots’ appearance and behavior (Hortensius et al., 2018) to enable nuanced and ecological valid training scenarios and improve medical simulation by ensuring inclusivity and important training variance (Conigliaro et al., 2020).

For a robot to simulate a human patient, clinical cues such as pain response, altered cognitive state, and emotional gestures need to be adequately captured for learners to recognize and perform the required actions for treatment (Moosaei et al., 2017). Hence the goal is to trigger the appropriate responses from users by the robots’ actions in simulated scenarios. This poses the challenge of determining sufficient facial movement capabilities for the different use-cases of the robot. Furthermore, trade-offs concerning scaling potential, robustness, and integration in existing equipment for clinical simulation training needs to be addressed. Since humanoid robots for healthcare learning applications require several anatomical features to enable clinical interventions and facilitate training and routine practice, the available design space is constrained. A multifaceted design problem is therefore inevitable, where both non-verbal communication and physical interventions are required to ensure ecologically valid training-scenarios. In addition, the facial movements of robots may introduce uncanny effects, aversion, misunderstandings, and expectation gaps (Kwon et al., 2016). To approach these challenges, there is a need for exploring and characterizing the capabilities of robots by common parameters such as AUs. Hence, we ought to explore the minimum viable expressive capabilities, and simultaneously uncover the expressive potential the robot can achieve given contextual design constraints. We have developed a humanlike robotic face prototype with facial actuation capabilities to highlight these challenges with potential solutions by exploring AI tools to evaluate and inform the current design. Using the robot as a sandbox we have generated a control model utilizing intuitive and high-level instructions by AU parameters instead of manual control and pre-programmed sequences.

### 2.3 The Prototype

**FIGURE 1:**
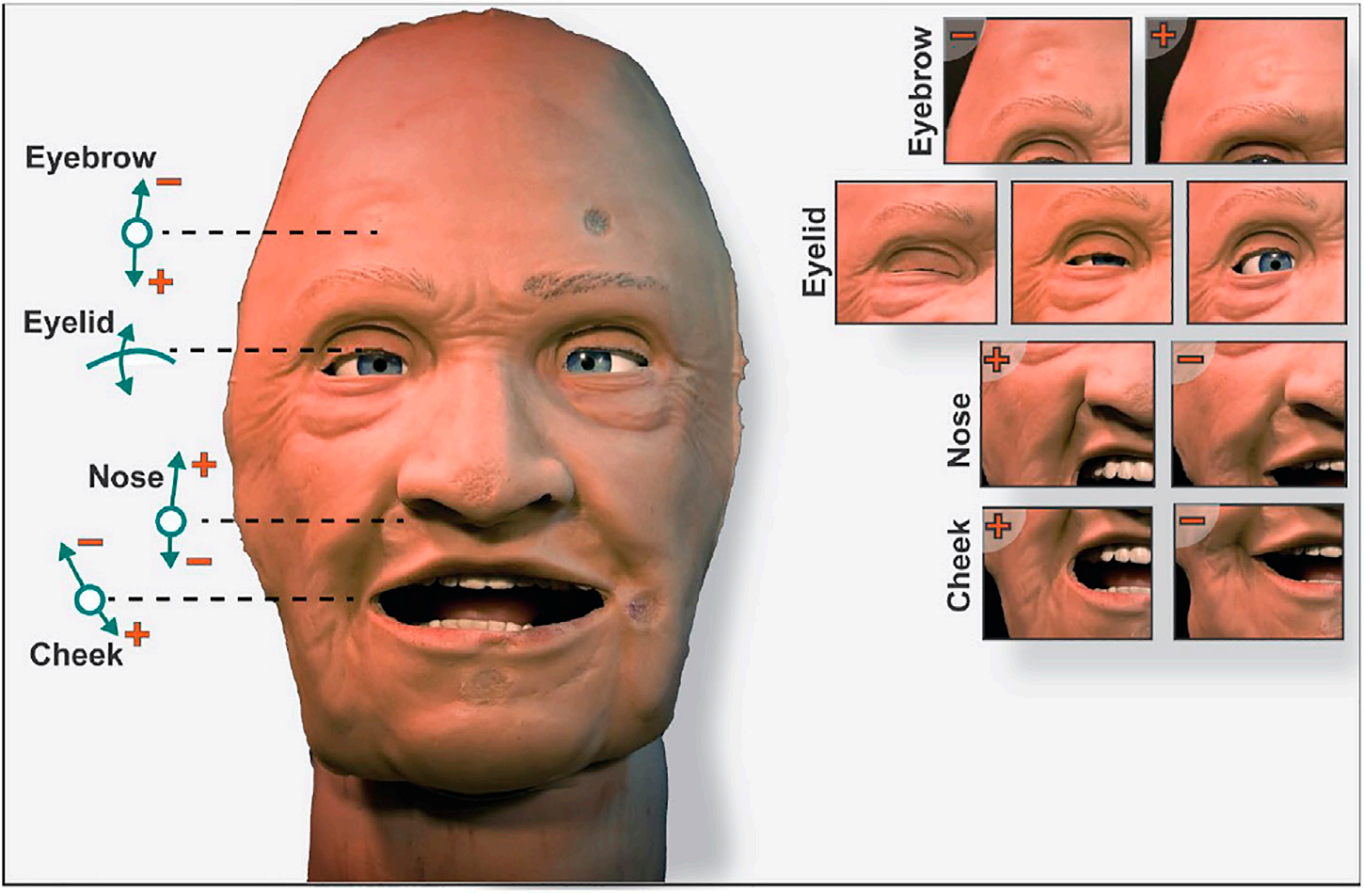
The robotic face prototype with indicated actuation points, direction of travel, and achieved face movements.

The robotic face prototype consists of a silicone-rubber skin with embedded skull-interface connectors, and a rigid skull chassis for mounting the actuators and providing structural support. The prototype also has eyelids that can open and close, with static eyeballs and a non-articulated jaw. An anatomically accurate (upper) airway with teeth and tongue is also a part of the assembly. Furthermore, the prototype has six individual actuation points located at the root of the nose, corner of the mouth (cheek), and eyebrows. The location of actuation points is set to accommodate anatomical artefacts such as the airway, eyes, and tactile landmarks to enable clinical interventions. This naturally limits available design space, and thus servo motors for actuation are connected through individual push and pull cable arrangements. The actuation is done by six inexpensive 9 g RC servos that are controlled by a 18-channel Pololu Mini Maestro servo motor controller (Pololu - Mini Maestro, 2021). Additionally, two servos are mounted behind the eyeballs with a bar linkage to actuate the eyelids. The prototype with indicated connection points, actuation modes, and direction of servo movement (positive and negative) are shown in Figure 1.

**FIGURE 2:**
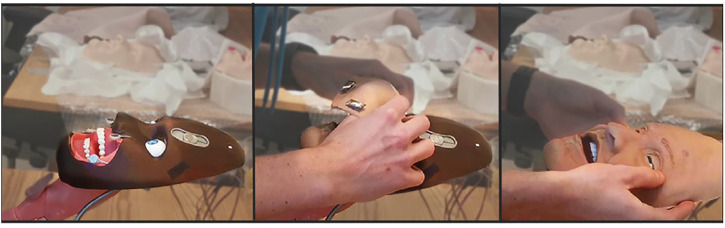
Modular face design for changing the visual appearance using interchangeable face skins.

To both alter the appearance and accommodate rapid and parallel design iterations, the robotic face and skull is designed with easily interchangeable outer skins, as seen in Figure 2. The current iteration of the facial skin portrays a geriatric male, where the proportions of the skull, relative distances between landmarks, and skull geometry remain fixed. Even though different characteristics pose facial anthropometric differences (Zhuang et al., 2010), the current use of (static) simulators with interchangeable skin appearances suggests the generalized hardware would enable a sufficiently wide design space to portray broad span of patients with similar anatomy. The skin is made from a highly flexible and low-density silicone rubber and is molded with variable thicknesses to simulate the tactility of facial tissue and muscles. The face to skull interface consists of mechanical snap connections embedded in the silicone, either interfacing the wire endpoints connected to the actuators, or as fixed anchoring to the skull. With this setup, various designs for the face skins can be explored, where flexible materials, appearance, geometry, and connector designs can be tested iteratively. Furthermore, alterations to the skull assembly can be tested with readymade face skins by changing actuator positions, connector positions, and structural geometry. This enables rapid design iterations and new prototypes to be generated and tested fast to address technical obstacles encountered through the development process.

Another important aspect of being able to easily swap face skins (apart from accommodating rapid design changes) is for the robot to portray various patients corresponding to the vast differences found in the human population, and thus increase training variance, inclusivity, and realism. This is especially important considering the robot is intended for healthcare learning applications (Conigliaro et al., 2020). As a result, the design objectives become more complex as the range of motion and connections between the face skin and skull should be tailored to the attached skin appearance. Since different appearances yield different face geometries, and thus mechanical properties of the artificial skin, it is not evident how each skin appearance would perform. Furthermore, as each appearance portrays a persons’ age, gender, cultural, ethnical, and other visual attributes, control of the robot, and subsequentially evaluating the output, becomes a challenge given both designer and user biases.

The conceptual prototype was developed to test assumptions and elicit technical requirements. The development has been highly iterative, and thus several prototypes have been built to answer technical questions, as well as being a manifestation of the idea that can be presented to users to gain important feedback (Auflem et al., 2019). However, the challenge of the subjective matter of facial cues and expressions is obtaining actionable and objective data to measure the performance of prototypes by multimodal evaluation (Moosaei and Riek, 2013; Ege et al., 2020). This is particularly challenging when evaluating expressive robots due to the resolution and fidelity of the presented prototype being perceived differently, especially when users are unaware of the current state of development. Furthermore, clear limitations such as the absence of a complete head, fully covered skin, and missing anatomical landmarks not addressed by the current prototype may influence the feedback (Houde and Hill, 1997). This makes the evaluation process both challenging, tedious, and costly. Alternative tools are, therefore, needed in the early development stages to quickly improve the design of robotic faces.

To rapidly evaluate the prototype with the current appearance, we propose using a facial behavior analysis toolkit for capturing human face attributes. We seek to obtain data on the possible numbers and intensities of AUs the robot can generate to inform the hardware design. Using this data could also accommodate the many control methods and input data we want to explore for the robot. This flexibility could be beneficial since it is not evident how we want to control the robot in simulated scenarios. For example, control instructions could be given based on face expression coding using FACS or tracking an operator for real-time reenactment (Nygaard et al., 2018). Also, in a medical context, we could create simulation sequences using video of real-life events and patient assessments. Therefore, since structured data is available on human face movements using AUs, we want to use AUs as framework for controlling and evaluating the prototype (Lalitharatne et al., 2020).

### 2.4 Open-Source Computer Vision and Machine Learning Methods

The facial behavior analysis toolkit OpenFace 2.0 (Baltrusaitis et al., 2018) consists of state-of-the-art computer vision algorithms for automatically detecting and estimating facial landmarks, head pose, eye-gaze, and AUs. The toolkit has been used for understanding and recognizing mental states and social signals in human subjects within numerous fields. With its successful utilization on human subjects, and because the algorithms are trained and validated on actual humans, we believe it can be used for improving the way robotic faces are developed and controlled. More specifically, if we can detect the AUs of the robotic face and map them to its actuation units, we can synthesize more humanlike facial expressions while alleviating the potential designer bias. The dynamic AU recognition framework in OpenFace 2.0 also employs a person-specific normalization step (Baltrušaitis et al., 2015), making it adaptable for individual faces instead of relying on generalization.

The behavior of the robotic face can be modeled by applying various ML methods using facial expression analysis for the input and actuation parameters of the mechanical face as output. Scikit-learn (Pedregosa et al., 2011) is an open-source ML library for Python that is simple to use and provides efficient tools for predictive data analysis. By randomly moving the face-servos and capturing the resulting AUs through OpenFace 2.0, the robotic face can autonomously learn its facial expressions using a generic Python application. The intensity of an expression can then be adjusted on a continuous range by applying regression analysis to enable a more objective way of controlling a robotic face. Combinations of AUs can thus be used to estimate facial expressions instead of manually adjusting the servo angles and subjectively assessing the resulting expression. The same ML methods can also be applied to different robot designs since a model can be trained for each actuator with the same AUs as input, where essential and redundant AUs are weighted accordingly through the optimization algorithm. Furthermore, the methods are not restricted by the number of actuators since the automatic AU capturing approach allows the creation of large sample sizes, although using many redundant actuators may cause some of them to influence each other in opposite directions, thus worsening the training data. A method to quantify the relevance of each actuator is therefore beneficial before creating the control models.

The ability of a robot to show specific emotional expressions can be further evaluated using Residual Masking Network (RMN) by Pham et al. (2021); a state-of-the-art ML model for facial emotion recognition. RMN has achieved the highest classification accuracy of 74.14% on the widely used Facial Emotion Recognition (FER-2013) dataset (Goodfellow et al., 2013), which includes 35,887 images of facial expression of humans in seven categories: anger, disgust, fear, happiness, sadness, surprise, and neutral. By using ML-based facial emotion recognition, we can evaluate the robot faster than finding experts or using multiple people through surveys.

## 3 Materials and Methods

### 3.1 Experiment Setup and Pipeline

**FIGURE 3:**
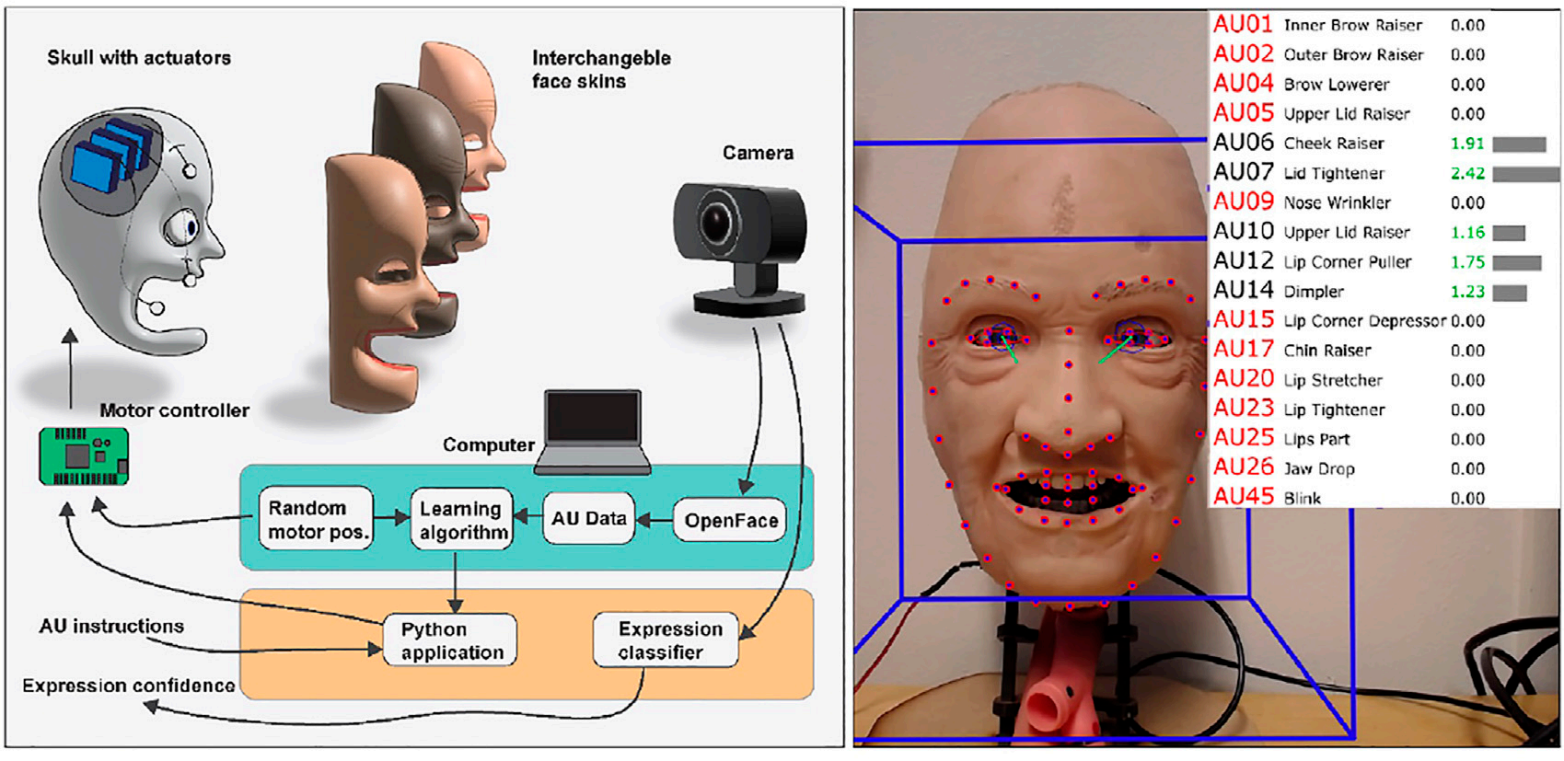
Pipeline for controlling and analyzing the robotic face, with an example frame captured and analyzed with OpenFace 2.0.

The pipeline for the proposed method is illustrated in Figure 3. It consists of two main parts: sending servo control instructions to the robotic face to change its appearance and capturing the characteristics of the resulting face using a camera and OpenFace 2.0. A Logitech C930e webcam was used for capturing frames of 1280 × 960 pixels to be analyzed with OpenFace 2.0 for predicting AUs. Both the webcam and Maestro servo controller were connected to a laptop through USB connections.

### 3.2 Data Collection

**FIGURE 4:**
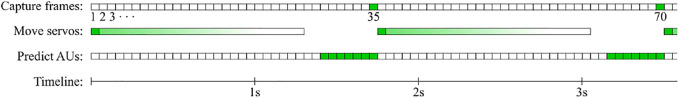
Illustration of the data capturing and analysis procedure. Each square represents one iteration, where green denotes the stages where data are recorded.

The range of motion for each servo was set manually through the Maestro control center and then normalized to a range between zero and one. The 17 AUs have intensity values ranging from 0 to 5, with 0 meaning the AU is not present, 1 representing presence at minimum intensity, and 5 representing presence at maximum intensity. Datasets containing AUs and servo positions were created by randomly moving the nose, eyebrow, and cheek-servos symmetrically along the vertical axis while recording the face through the webcam to extract AUs at a rate of approximately 20FPS. Due to the framerate of the webcam being affected by several factors including processing power and lighting (exposure), the framerate fluctuated slightly during real-time analysis. The AU estimations also fluctuated accordingly during static facial expressions. Therefore, based on a few initial tests, we found that capturing every 35th frame and taking the average AU values of the seven preceding frames reduced the variance and improved robustness while allowing the face servos to settle for each random position. The data collection and analysis sequence are illustrated in Figure 4.

Since OpenFace uses person-specific normalization, which assumes that a neutral facial expression is present in most of the analyzed frames in a video sequence, we need to find the frequency of randomly moving the servos and returning them to a neutral position to get the most consistent AUs. The effect of AU normalization was therefore analyzed by adjusting the frequency of returning the face to a neutral position (servos at 0.5) and capturing the combined mean of the resulting AUs to observe the overall variance. Based on an appropriate frequency of moving the face to a neutral position, a final dataset was captured where 500 frames were extracted containing random servo positions and the corresponding AUs. The datasets consist of the 17 AUs shown in Figure 3 as independent variables and the three symmetrical servo positions shown in Figure 1 as the dependent variables, excluding the eyelid.

### 3.3 Correlation Analysis

The Pearson correlation coefficients between every pair of variables were calculated using the “pandas.DataFrame.corr” module in Python and subsequently visualized using a correlation matrix. In addition to showing how the servos relate linearly to AUs, the correlation matrix can also indicate if AUs correlate to each other. If, for example, two AUs have a strong correlation, we can deduce that the current servo configuration cannot distinguish the respective AUs, thus providing valuable feedback to the development process. Redundant actuators can be discovered by detecting a corresponding lack of affected AUs, which can be disconnected to reiterate the process and analyze its effect. In addition to analyzing the current robot design, the correlation matrix is a tool for the development process to continuously discover possible improvements to the design and make sure the training data for the control model is appropriate.

### 3.4 Modeling and Evaluation

We trained several regression models for predicting the servo positions with AUs as input. A supervised learning approach can be used since the input and output samples are collected directly through the pipeline. A few common supervised ML methods, namely linear regression (LR), ridge regression (RR), support vector regression (SVR), and multilayer perceptron (MLP) were used through the scikit-learn library (version 0.23.1) to test how different linear (with and without regularization) and non-linear learning algorithms affects performance. Exploring multiple ML methods without requiring considerable time is feasible if the number of samples is within a few thousand. The dataset was split into a training set (80%) and a test set (20%). Grid search with 5-fold cross-validation, provided by the GridSearchCV module from the scikit-learn library, was used when training models containing hyperparameters, as shown in Table 1. A random state value of 42 was used for the MLP model to preserve reproducible results. The best model for each servo was selected based on the lowest root-mean-square error (RMSE) and then evaluated with the test set.

**TABLE 1:** Models and parameter values used for the grid search training procedure.

Model	Hyperparameters	Parameter values
Linear regression	—	—
Ridge regression	Alpha	0.01, 0.1, 1, 10, 100
SVR	C	0.1, 1, 10, 100, 1,000
	Gamma	1, 0.1, 0.01, 0.001
	Kernel	Rbf, linear, sigmoid, poly
MLP	Hidden layers	1, 10, 25, 50
	Activation function	Identity, logistic, tanh, relu
	Solver	Lbfgs, sgd, adam
	Alpha	0.00005, 0.0005
	Learning rate	Adaptive
	Max iterations	5,000
	Random state	Numpy.random.RandomState (42)

Learning curves are also presented to assess the effect of training set size and to discover potential bias and variance in the data. The number of training samples was incremented by 15 and subsequently trained using the optimal parameters found from GridSearchCV and evaluated with 100 validation samples, where the RMSE for both sets was reported in the learning curves.

The servo positions for six expressions were then estimated using the best models by maximizing the relevant AUs based on FACS and setting the others to zero. The resulting facial expression of the robot was subsequently captured with the webcam and evaluated using RMN. Although the RMN model uses a softmax function for its output and consequently returns a confidence score for each of the seven output categories, we only report the top two predictions. Additionally, to demonstrate an alternative method for dynamically controlling the robot, we capture the AUs from a person in real time as input for the models to predict the servo positions, i.e., using mimicry to control the robotic face. This presented pipeline and experiments can be seen in a video added as Supplementary Material.

## 4 Results

### 4.1 Effect of Action Unit Normalization and Correlation Analysis

**FIGURE 5:**
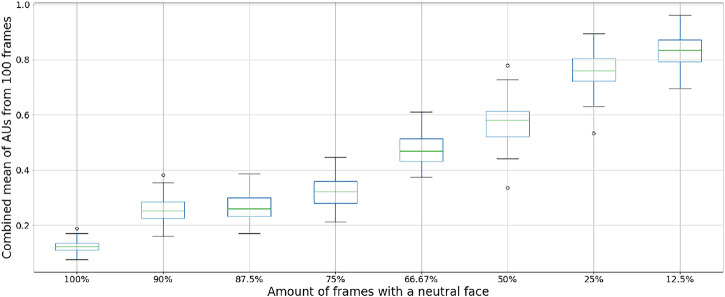
Distribution of combined AU intensity values based on the frequency of a normal face analyzed with OpenFace 2.0.

**FIGURE 6:**
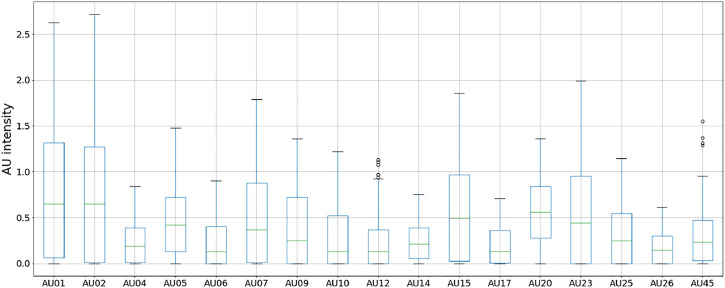
Distribution of AU intensities captured from the final 500 random face-servo positions.

The distribution of the average AU intensities based on the frequency of setting the robotic face to a neutral position is shown in Figure 5. As expected, displaying the neutral face constantly (100% of the frames) results in the lowest AU values with the least variation. The variation increases when introducing random positions in 10%–25% of the frames (90%–75% neutral faces), while the AU values increase substantially when only 66.7%–12.5% of the frames contain a neutral face position. A trade-off between speed and the effect of person-specific normalization was made when capturing the final dataset, where 75% of the frames contained neutral positions. Thus, we captured a total of 2,000 samples, for which 500 contained the random servo positions included in the final dataset. The distribution of intensities for each AU for the final dataset is shown in Figure 6.

**FIGURE 7:**
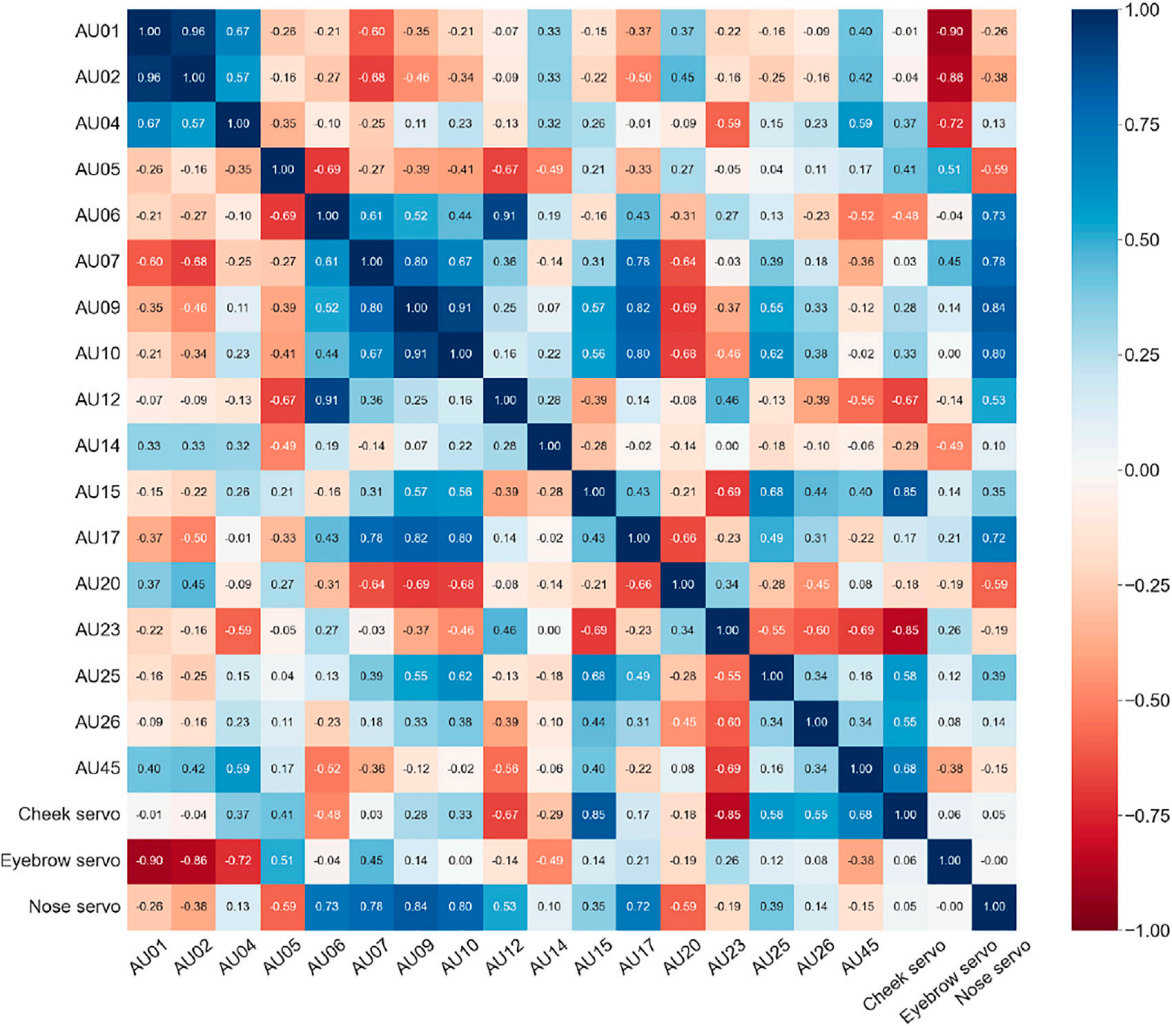
Correlation matrix showing the Pearson correlation coefficients between AUs and servos. The blue to red gradient indicates the strength of positive and negative correlations, respectively.

The correlation matrix in Figure 7 shows the Pearson correlation coefficients between all AUs and servos. Every correlation coefficient higher than 0.1 in absolute value is statistically significant (*p* < 0.05). Furthermore, each AU has a moderate to strong correlation with at least one of the servos, with AUs 5, 14, 20, 25, and 26 having the weakest (absolute values between 0.49–0.59), and AUs 1, 2, 9, 10, 15, and 23 having the strongest (absolute values above 0.8). While the strong correlation between AUs 1 and 2 is expected since they represent the eyebrows, the correlation for AUs 7, 9, and 10 with AUs 17 and 20 is less expected and may indicate a limitation in the current servo configuration.

### 4.2 Regression Analysis and Evaluation

The results on the test set after training and validating each model are shown in Table 2. Each method achieves low RMSE, with MLP having the lowest for each servo. Given the large number of independent variables, it is expected that SVR and MLP can capture some of the non-linear effects of the input and thus achieve lower errors.

**TABLE 2:** RMSE on test set for best models. Lowest values are highlighted in bold.

	LR	RR	SVR	MLP
Cheek servo	0.054887	0.054788	0.054162	**0.040817**
Eyebrow servo	0.073310	0.073149	0.071086	**0.067251**
Nose servo	0.084295	0.083979	0.062116	**0.059183**

**FIGURE 8:**
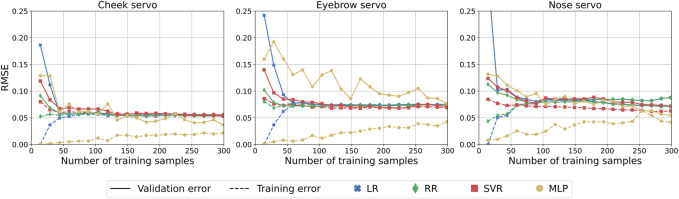
Learning curves showing the effect of training samples on validation and training error.

Using the parameters found for the best models through cross-validation and grid search, we retrained each model using increments of 15 training samples, resulting in the learning curves shown in Figure 8. Here we can observe that relatively few training samples were needed to converge to an optimal solution for LR, RR, and SVR models. Low variance is observed by the comparable training and validation accuracy, indicating that no more than ∼60 training samples are needed. The models do not appear to be affected by high bias given the low errors for both datasets, demonstrating that using AUs as features for predicting the servo positions is feasible and does not result in overfitting.

## 5 Interpretation of Results and Experimental Evaluation

Based on the data we have generated from using OpenFace 2.0 and the results from training a control model for the robot, this section presents how we can gain valuable information and insights using the proposed tools when developing humanoid robots. Sections 5.2, 5.3 demonstrate how we can use the control model to generate facial expressions and dynamic motion through real-time reenactment.

### 5.1 Evaluating Robot Face Movement Capabilities Using Generated Action Unit Data

To obtain actionable data for improving the current design, we reviewed the AU intensities and respective correlations between variables seen in Figures 6, 7. The distribution of AU intensities shown in Figure 6, indicates that only subtle expressions are obtainable as none of the captured AUs reach high levels ranging from 0 to 5. However, while the overall intensities of the recorded AUs are low, we can distinguish between the recognized output movements that are obtainable and the ones that remain idle given the current setup. We can further investigate the movement capabilities by looking at the correlation matrix in Figure 7, which shows that each servo has several strong correlations with action units. Based on these observations we can identify, and investigate, desirable AUs with weak intensities for improving the range of motion and location of actuation points. Furthermore, we can identify desirable correlations, such as servo to AU correlations, and inseparable AUs limiting isolated face movement given the current design.

Figure 7 shows a strong correlation between AU01, AU02, and the eyebrow servo. This is expected as they correspond to the inner and outer brow raiser, respectively. Their correlation with the eyebrow servo means that moving the servo upwards (in the negative direction) is identified as raising of the brows. However, differential control of these AUs is not possible, meaning that both the outer and inner brow will move simultaneously. Looking at the other action unit related to brow movements, we find that AU04 also correlates negatively with the eyebrow servos, which may seem counterintuitive since an increase in the eyebrow servo (Figure 1) should result in an increase in AU04 (brow lowerer). However, AU04, also known as the corrugator and depressor supercilli (Bartlett et al., 2005), represents the constriction of the area between the eyebrows in addition to brow lowering, which can happen simultaneously with AU01 and AU02. This means brow lowering is obtainable, although with a constrained path and weaker correlation indicating that a greater travel distance is desired to fully achieve this actuation mode. This is also highlighted in Figure 6, where the obtainable intensity of AU04 is significantly lower than that of AU01 and AU02, indicating that both travel and location of the brow connection point should be re-evaluated to enhance the brow lowering capability. Hence, we can improve the balance between the observed action units through physical design changes to create a model with more realistic brow movements.

In the mouth and nose area, we can observe a correlation between the action units AU09 (nose wrinkler) and AU10 (upper lip raiser), and between AU06 (cheek raiser) and AU12 (lip corner puller). These AUs also correlate respectively to the nose and cheek servos. This indicates some of the dependent movements that are inseparable given the current design, caused by the artificial facial skin being a single deformable structure. Without additional anchoring of the face skin, deformations will be transferred throughout the elastic material and thus cause deformations ahead of the interface point along the line of travel in the face. While these connected movements are also found naturally in expressions by human faces, additional anchoring or actuation points would be required to separate them and enhance the movement capabilities of these AUs. Other correlations such as AU07 (lid tightener) and AU17 (chin raiser) with both the nose servo and to other action units, is also worth investigating. Since the eyelids are not actuated in the presented results, the intensity of AU07 is surprisingly high, suggesting additional anchoring around the eyes could be required. AU17 on the other hand, has a low intensity as seen in Figure 6, indicating that despite correlation with other AUs, the observed movement might be negligible. These findings suggest that either additional anchoring or additional actuation points could be beneficial to mitigate inseparable movements considering lower lips, jaw, and lower eyelid, and thus achieving a broader span of individual face actions. These are tradeoffs that should be evaluated alongside the required movement capabilities, as additional actuation modes would require additional actuators.

We can also identify which AUs are least represented in Figure 6, and review their correlation to the servos and other AUs in Figure 7. Here we highlight AU06 (cheek raiser), AU10 (upper lip raiser), AU12 (lip corner puller), AU14 (dimpler), AU17 (chin raiser), AU25 (lips part), and AU26 (jaw drop). These action units concern areas of the face located distant to the actuation points, indicating that we can only obtain movement in these areas by targeting nearby action units with a stronger presence in the current prototype. This is however not the case for AU25 and AU26, that shows modest intensities with few and weak correlations. Because the prototype has a fixed jaw and no lower lip actuation, these findings match our assumption that little movement should be obtained in these areas. These findings show that utilizing action unit data to describe and evaluate robotic faces to obtain actionable design input is purposeful.

### 5.2 Predicting Servo Positions Using Action Unit Instructions and Residual Masking Network

Six robot expressions were generated by predicting each servo position using the MLP models, with the relevant AUs maximized for the input. An image of each expression was then analyzed with RMN, resulting in the predictions shown in Table 3.

**TABLE 3:** Generated face expressions with RMN predictions.

Expression	Anger	Disgust	Fear	Happy	Sadness	Surprise
Maximized AUs	4, 7, 23	9, 15	1, 2, 4, 5, 7, 20, 26	6, 12	1, 4, 15	1, 2, 5, 26
Resulting expression	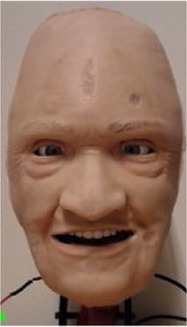	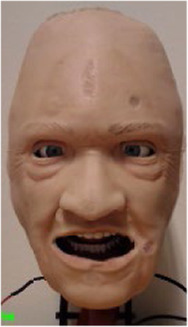	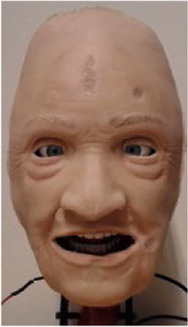	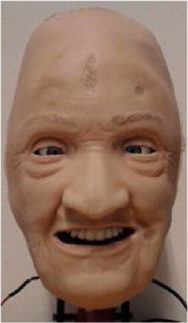	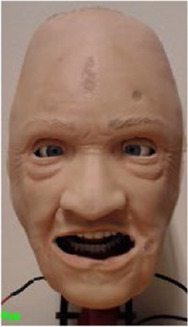	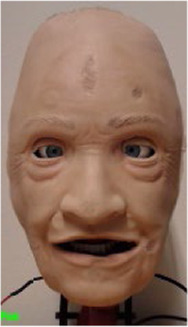
Top RMN predictions with confidence	Happy: 0.954	Angry: 0.401	Surprise: 0.900	Happy: 0.433	Surprise: 0.565	Surprise: 0.901
	Surprise: 0.046	Surprise: 0.368	Angry: 0.064	Surprise: 0.421	Angry: 0.333	Happy: 0.099

By sending instructions based on the FACS, we obtained a performance indicator on how the rendered expressions compare to sampled face expression images in the utilized dataset. These results indicate whether we can control the robot through AU instructions and render expressions that are recognized by ML. Furthermore, we can inspect the least successful expressions and correlate this back to the obtained AU data we have previously reviewed. Amongst the top confidence scores, only the Surprise and Happy expressions were correctly predicted. Disgust and sadness expressed by the robotic face are predicted similarly by RMN, both resulting in Angry and Surprise predictions with low confidences, which may be explained by the few representative AUs while sharing AU15. The limitations of the eyebrow actuation and the eyelids (not being active) could also be critical, since they impact the AUs for both anger, fear, and sadness expressions. The overrepresentation of Surprise predictions from RMN might be due to the underestimated importance of the eyelids, affecting AU5 (upper lid raiser), in combination with the non-articulated jaw affecting AU26 (jaw drop). The static jaw also limits obtainable AUs concerning mouth shapes and movement, which may explain why the RMN is less successful at predicting the negative expressions (where AUs around the mouth is particularly important). This suggest that additional actuation around the mouth and jaw is desirable to achieve a broader span of obtainable emotion expressions.

### 5.3 Real-Time Reenactment Using Recorded Action Units From Human Actor as Input

**FIGURE 9:**
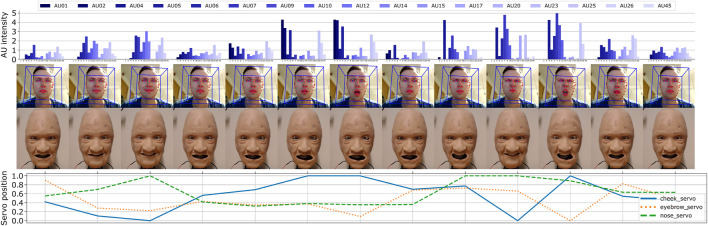
Real-time reenactment using recorded AUs from a human actor to predict the servo positions.

The MLP models were further utilized to experiment with real-time and automatic control of the robotic face. Here, we attempted to mimic facial movements by capturing AUs from a human actor using the same toolkit as previously described (OpenFace 2.0). By tracking the actors face through a webcam, we obtained and sent a stream of unfiltered AU intensities to the trained MLP models which then predicted and adjusted the servo positions in real time. A few frames from the face-tracking and the corresponding robot actuation are shown in Figure 9, including the raw input data (AU intensities) and the predicted output (servo positions). Although we can extract AUs at 20FPS, our application recorded at roughly 12FPS due to storing each captured frame as images, both of the human actor and robot simultaneously, where the robot face was updated at approximately 2FPS due to the delay in moving the servos.

## 6 Discussion

### 6.1 Using Open-Source Software for Robotic Face Development

Our results demonstrate the use of open-source computer vision and ML methods as tools for supporting the development and evaluation of robotic faces. With careful consideration of how OpenFace 2.0 is utilized, such as the processing sequence shown in Figure 4 and the effect of person-specific normalization, it is possible to reliably capture and utilize AUs to better understand the capabilities and limitations of a robotic face. Furthermore, since the approach is not reliant on the robot’s specific characteristics or control parameters, it can be applied to any robotic face resembling a human.

We have also shown that AUs can be used to predict the servo positions for objectively controlling the robot, with errors as low as 0.041–0.067 RMSE. However, in our specific case, the learning curves and the correlation matrix reflect some limitations in the captured data, indicating the absence of dispersed and complex combinations of generated AUs. Since our robotic face can only produce a limited set of variability on AU combinations and intensities, the models may show low errors on test data using relatively few training samples while being unable to predict the servo positions appropriately for entirely different combinations of AUs never before seen. Suppose the goal is to simulate specific emotions (combinations of AUs). In that case, it is essential to focus on the correlation between AUs and reduce their interdependency through physical design choices, thus allowing more complex AU combinations to occur. We were nonetheless able to generate facial expressions by combining relevant AUs, as demonstrated in Table 3, and using mimicry to control the robot, showing generalizable tendencies of the trained ML models. These results are especially promising when considering the few actuators used in the robot design.

While the RMN model provides a quick and easy way to evaluate the expression of a robotic face, it may not be an accurate representation of how experts or the public perceives it. The model is trained on the FER-2013 dataset with labels corrected by humans, which may introduce biases, as well as having an unbalanced distribution of expressions. Interestingly, the human accuracy was roughly 65% on FER-2013, according to Goodfellow et al. (2013), which is about 9% lower than RMN. Due to various factors such as location, perceived gender, and age affecting people’s subjective judgment (Moosaei and Riek, 2013), using an ML-based expression classifier may provide comparable accuracies while being more efficient in terms of time and resources. People should therefore be included in the evaluation stage when the robot is sufficiently developed and can be applied in the context it was created for.

### 6.2 Outlook, Further Work, and Limitations

Utilizing accessible AI tools has proven valuable in generating and evaluating a functional robotic face prototype to be further tested with users in intended use cases. The objective insights presented were enabled by the speed and flexibility of generating AU data to inform the design, train a specific control model, and evaluate performance using the FER-2013 dataset. Based on the acquired results, we see potential for this method to assist further development steps. Firstly, from the iterative nature of early-stage development, insights and elicited requirements (from users) can be addressed by altering or generating new prototypes. This is accommodated by quickly and cheaply generating a control algorithm and obtaining objective evaluation not restricted by hardware capabilities or appearance traits. Secondly, expanding the portfolio of facial appearances is supported by the flexibility and valuable feedback provided in the AU data. This implies that each appearance can be tested and evaluated fast, and a model can be trained to utilize the actuation capabilities of the given robot face. These points highlight that rapid and objectively informed design iterations are possible, even when addressing complex and multifaceted problems, such as expressive humanoid robots. We showcase this by the results and insights obtained for the current iteration of our robotic face intended for medical simulations.

The presented results in this study indicate that the current robot cannot generate strong AU intensities. This finding is further scrutinized by relating the AU data to the hardware setup and suggesting how to improve the design by looking at the AU and servo correlations and the intensity of AUs in the generated dataset. The weak AU intensities and strong correlations could suggest that in addition to the range of motion, more human artifacts such as particular wrinkles, textures, and distinct landmarks such as marked eyebrows could amplify the obtainable AUs. However, the current performance of the robot is still sufficient to train a control model, as previously discussed, and the robot can mimic a human actor based on unfiltered AUs recorded using a fast and flexible pipeline. These results are promising as ours and other robotic faces can benefit from the presented support tools to inform and speed up the design process. Development tools are also essential as the complexity of the robotic setup increases, where both manual operation and hardware evaluation become more challenging and time-consuming. Thus, the data generated in the form of both descriptive intensities of AUs and correlations between actuation modes and generated output is purposeful when pursuing more complex behaviors and expressions rendered by robotic faces. In addition, our approach can address the non-linear relations between obtained AUs and actuation modes which is essential as increasing actuation points would increase interdependencies and complex behavior since the skin is a single deformable structure. Concerning more complex hardware setups, the generated data and correlations between input and output parameters could be a helpful tool in addressing actuators not correlating to detected action units or even interfering with other actuation modes limiting the expression output of the robot. Our approach should be applied on robot faces with varying degrees of freedom to further validate these potential advantages.

Since OpenFace 2.0 is essentially made for analyzing the facial behavior of humans (and now also of robots), it can additionally be used in the control system of the robot itself to analyze humans during interactions and adjust its non-verbal communication approach accordingly. The utilization of OpenFace 2.0 can also be scrutinized to advance the method further by incorporating facial landmark detection to measure facial deformations or tracking head pose and eye-gaze to analyze gaze behavior, which is essential for improving human-robot interactions (Abubshait and Wykowska, 2020). An approach for automatically finding the most neutral expression of the robot should also be explored to enhance the effect of person-specific normalization and potentially increase the range of AU intensities.

As a proof of concept, the expressive capabilities of the robotic face is tested by manually sending AU instructions to a trained control model. Static emotional expressions are difficult to evaluate, even for humans, so investigating dynamically changing expressions is interesting (Mollahosseini et al., 2019). Hence, the transition between expressions, actuation speed, and mechanical noise are essential parameters. Since AUs can be used as a transferal unit between humans and robots, facial responses from humans can be automatically captured to simplify the dynamic control of the robot. This approach could further be leveraged to generate models to automatically control the speed, onset, and offset of different expressions. Other examples for controlling the robot using AUs include real-time reenactment by operator, obtaining AU data sequence from video samples, or allowing users to generate custom expressions by either saving, mirroring, or manually adjusting AUs performed by the robot. The experiment using an actor for real-time reenactment of face movements suggests these possibilities to be promising. However, even though OpenFace 2.0 can analyze AUs in real time, our current robot design is limited by the actuator control unit moving the servos one at a time. By implementing a controller that can control several servos in parallel and with higher power output, this bottleneck can be reduced or potentially removed to increase response times and enable more nuanced motions during dynamic interaction.

While we in this paper discuss the applicability of using fast and accessible AI-based software to analyze robotic face movements, we acknowledge the importance of human user-interaction and evaluations in this setting (Moosaei and Riek, 2013). Human perception is particularly important for robots to be used in medical training scenarios, as the learning effects of having facial movement capabilities, and users’ ability to respond to these, is not possible to deduce in any other way. This is also supported by users’ limited ability to evaluate expressions rendered by alternative mediums or agents, suggesting that a physical robot is required to get further design inputs (Hofree et al., 2018). Concluding necessary design alterations, we want to pilot the robotic face in training scenarios and explore interactions and potentials for having expressive capabilities. Furthermore, as medical simulation is performed in teams, understanding the implications expressive cues of robots could pose on team dynamics is crucial. Therefore, utilizing the robotic face and control model to elicit requirements for expressions and facial responses that can enhance medical simulation scenarios is essential for future development.

The tests and insights showcased in this paper are obtained using only the face portrayed in the current prototype, and thus, limitations for using other appearances could be encountered. For example, how realism and fidelity concerning the visual appearance of the prototype influences AU data generated by OpenFace 2.0 and distort the confidence scores from RMN. This brings to question how closely the prototype needs to resemble a generalized human face. Furthermore, to explore visual edge-cases of the robotic face, appearance traits such as proportions, complexion, texturing, hair, tattoos, or scarring could be investigated. Further work would also be required to evaluate the effects of age, gender, and ethnicity, evaluate the robustness of the tools utilized, test for biases in the utilized data, and expand the suite of available appearances for the robotic face. It is also not evident how a generalized hardware setup accommodates the various facial characteristics as this would suggest anthropometric differences concerning size, proportions, and landmark location. However, we believe that a standardized setup would enable a sufficient design space to explore the potentials and limitations of switching the appearance of humanoid robots such as this one. As this is a crucial aspect of ensuring inclusivity and training variance in medical simulation, we see the approach utilized in this paper as effective for enabling faster prototyping iterations when developing humanoid robots.

## 7 Conclusion

We have presented methods utilizing open-source AI tools for supporting the development and evaluation of robotic faces. First, dynamic AUs of the robotic face were automatically captured through OpenFace 2.0 during random movements to find correspondence between facial attributes and the servo configuration. The correlations between AUs and servos provided objective feedback on the possibilities and limitations of the robot design. Next, a control model for the robot was developed by estimating the relationship between AUs and servo positions through regression analysis, enabling facial expressions to be rendered using AUs as input. We then evaluated the simulated expressions using a classifier trained on a large dataset of human facial expressions, providing additional assessment opportunities for the robotic design and control model. The methods have proven to be beneficial during early-stage development to rapidly gain actionable insights, in addition to being low-cost and easy to use.

## Data Availability

The raw data supporting the conclusion of this article will be made available by the authors, without undue reservation.
